# Associations between long-term exposure to air pollutants and mortality risk of critically ill patients: a multi-center cohort study in central China

**DOI:** 10.1186/s13613-025-01527-y

**Published:** 2025-10-21

**Authors:** Lu Ye, Chen Li, Kun Qin, Liang Xu, Ping Jin, Zhanpeng Wang, Cong Zhang, Chun Yin, Yaolin Liu, Zhicheng Fang, Jingjun Lv, Peng Jia

**Affiliations:** 1 Department of Emergency, Renmin Hospital of Wuhan University, Wuhan, China; 2 International Institute of Spatial Lifecourse Health (ISLE), Wuhan University, Wuhan, China; 3 School of Resource and Environmental Sciences, Wuhan University, Wuhan, China; 4 Department of Emergency and Critical Care Medicine, Wuhan Wuchang Hospital, Wuhan University of Science and Technology, Wuhan, China; 5 Department of Intensive Care Unit (ICU), The Second Clinical Medical College, Jingzhou Central Hospital, Yangtze University, Jingzhou, China; 6 Division of Natural and Applied Sciences, Duke Kunshan University, Kunshan, China; 7 Department of Emergency, Taihe Hospital, Hubei University of Medicine, Shiyan, China; 8 Renmin Hospital (First School of Clinical Medicine), Wuhan University, Wuhan, China

**Keywords:** ICU, Air pollutant, Mortality, Critically ill patient, WQS

## Abstract

**Study objective:**

Air pollutants have been known as the most persistent environmental risk factors of all-cause mortality in general populations. However, few studies focused on such associations in critically ill patients who usually suffer from multiple comorbidities and even organ dysfunctions, and thus have lower resistance to external risk factors. For the first time, this study examined associations between long-term exposure to air pollutants and mortality risk of critically ill patients, also relative contribution of each pollutant to their joint health effect.

**Methods:**

The 7,562 critically ill patients admitted to intensive care units (ICU) in a Hubei Province Medical Treatment Alliance in China were used in this study. Patient’s death within 28 days after ICU admission was used as the outcome. Daily concentrations of air pollutants, including PM_2.5_, PM_10_, NO_2_, SO_2_, O_3_ and CO, over their residence were estimated at a spatial resolution of 1 km by a newly developed multi-output LightGBM model, with better accuracy than all existing products. Logistic regression models were fit to estimate associations between individual air pollutants and mortality risk. Weighted quantity sum (WQS) regression was used to estimate relative contribution of each air pollutant to their joint effect on mortality risk.

**Results:**

The 7,222 patients were included in the study and had a mortality rate of 39.1%, with about half staying in ICU for ≤ 6 days. An increased risk for mortality was associated with a higher concentration of PM_2.5_ (OR = 1.007 [1.003, 1.011]), PM_10_ (OR = 1.002 [1.000, 1.004]), NO_2_ (OR = 1.020 [1.015, 1.024]), SO_2_ (OR = 1.025 [1.001, 1.050]), O_3_ (OR = 1.005 [1.001, 1.009]), and CO (OR = 4.336 [2.952, 6.457]). These associations varied across subgroups. For example, stronger associations were observed in males (PM_2.5_: OR = 1.010 [1.005, 1.015], PM_10_: OR = 1.004 [1.001, 1.007], NO_2_: OR = 1.026 [1.021, 1.032], and CO: OR = 6.224 [3.867, 10.019]), smokers (SO_2_: OR = 1.132 [1.078, 1.189], O_3_: OR = 1.014 [1.006, 1.022]), alcohol drinkers (SO_2_: OR = 1.147 [1.082, 1.215], O_3_: OR = 1.020 [1.010, 1.029]), and patients with a SAPS II of > 33 (SO_2_: OR = 1.168 [1.130, 1.207], CO: OR = 3.557 [2.165, 5.843]). The largest contribution to their joint effect on mortality risk was from O_3_ (43.8%), followed by NO_2_ (25.1%), CO (20.9%), PM_2.5_ (9.1%), SO_2_ (1.0%), and PM_10_ (0.1%).

**Conclusion:**

Exposure to air pollutants was positively associated with the mortality risk of critically ill patients, with O_3_ being the main contributor to their joint effect. The findings would help multiple stakeholders, including researchers, physicians, and policy-makers, better understand health effects of air pollutants on critically ill patients, also serve as justifications for facilitate environmental justice and health equity.

## Introduction

Air pollution is a major public health concern and an important cause of premature death worldwide [1]. It has been estimated that around 4.2 million premature deaths per year could be directly attributable to air pollution, which has become the 4th leading risk factor of mortality across the globe [2]. Global awareness of and research efforts on health effects of air pollutants have been increasing in recent years. Air pollutants that were found harmful to human health in existing studies mainly include particulate matter with aerodynamic diameter < 2.5 μm (PM_2.5_) and < 10 μm (PM_10_), nitrogen dioxide (NO_2_), sulfur dioxide (SO_2_), ozone (O_3_), and carbon monoxide (CO) [3]. Exposure to those air pollutants has been associated with a multitude of specific diseases, including respiratory, cardiovascular, neurological, allergic, and mental health outcomes [4–6].

Patients, usually with at least one comorbidity associated with air pollutant exposure, are more vulnerable than the general populations regarding negative health effects of air pollutants [7]. Among them, critically ill patients, usually suffering from multiple comorbidities and even organ dysfunctions and thus having lower resistance to external risk factors, may be the most vulnerable population and disproportionately affected by air pollutants [8]. On one hand, deaths in critically ill patients may mainly be attributed to serious outcomes, such as cardiopulmonary failure, the higher risk for which was associated with longer exposure to the higher concentrations of air pollutants [9–11]. On the other hand, critically ill patients tend to experience a systemic inflammatory response, which could be exacerbated by longer exposure to the higher concentrations of air pollutants [12]. However, no evidence is available on health effects of long-term exposure to air pollutants among critically ill patients.

To fill the aforementioned gaps, this study aimed to examine associations between long-term exposure to air pollutants and mortality risk of critically ill patients, as well as relative contribution of each air pollutant to their joint effect on mortality risk. The findings are expected to help multiple stakeholders, including researchers, physicians, and policy-makers, better understand health effects of air pollutants on critically ill patients. They would also serve as justifications for future intervention design and policy making, to alleviate air pollution and improve environmental justice and health equity.

## Methods

### Study subjects

The 7,562 critically ill patients admitted to intensive care units (ICU) in a Hubei Province Medical Treatment Alliance in China were used in this study, including the Hubei Province General Hospital (i.e., Renmin Hospital of Wuhan University), Guanggu Central Hospital (East Campus of Renmin Hospital of Wuhan University), Taihe Hospital (i.e., Affiliated Hospital of Hubei University of Medicine), Wuhan Wuchang Hospital (i.e., Affiliated Hospital of Wuhan University of Science and Technology), and Jingzhou Central Hospital (i.e., Affiliated Hospital of Yangtze University). Two periods of time were separately selected before and after the implementation of COVID-19 containment measures: from August 2018 to August 2019, and from May 2021 to December 2023. The electronic medical records of all patients aged ≥ 18 years admitted to the ICU of the five hospitals during the two periods were included in this study. This study was approved by the Clinical Research Ethic Committee of Renmin Hospital of Wuhan University (WDRY2023-K067) and fully complied with the Helsinki Declaration.

### Dependent variables

The 28-day all-cause mortality after being admitted in ICU has been generally considered the most appropriate and meaningful endpoint of critically ill patients, and widely used in clinical trials [13–15]. Therefore, death within 28 days after ICU admission was used as the outcome in this study.

### Independent variables

The exposure to air pollutants was measured as the 6-month mean concentrations of six pollutants (i.e., PM_2.5_, PM_10_, NO_2_, SO_2_, O_3_ and CO) prior to the date of hospital admission over the patients’ residence, averaged from the daily products of these pollutants. The daily concentrations of PM_2.5_, PM_10_, NO_2_, SO_2_, O_3_ and CO were modeled at a spatial resolution of 1 km by a multi-output LightGBM model, integrating a multi-output regression algorithm with the LightGBM (an open-source framework that implements the Gradient-Boosted Decision Tree algorithm), on the basis of multiple data sources including ground-based measurements, satellite-derived data, auxiliary data (e.g., meteorological, land surface, socioeconomic data), and spatial and temporal features [16]. 

### Covariates

The covariates adjusted in the models included sex (male, female), age (< 65, ≥ 65 years), smoking (yes, no), alcohol drinking (yes, no), and the Simplified Acute Physiology Score (SAPS II) (≤ 33, > 33; halved by the median) [17]. SAPS II is a quantitative score reflecting the severity of disease in patients, calculated based on the scores of 17 variables, including age, ICU admission type, presence of 3 comorbidities, and 12 physiological variables [18]. According to the conditions of the patient, each variable scored a value of 0–26, and the total SAPS II score ranged from 0 to 163, with the higher score indicating the greater severity of disease. For the variables with multiple measurements during the 24 h after ICU admission, the worst measurement from clinical and laboratory records was assigned to each of them.

### Statistical analysis

The characteristics of the subjects were presented as the number and percentage for categorical variables, and as the mean and standard deviation (SD) for continuous variables. χ^2^ tests (for categorical variables), *t*-tests (for continuous variables), and Mann-Whitney *U* tests (for variables denoted by the median [interquartile range, IQR]) were conducted to assess the significance of differences in the subjects’ characteristics. Logistic regression models were fit to estimate associations between individual air pollutants and mortality risk, expressed as odds ratio (OR) and 95% confidence interval (CI). Subgroup analysis was conducted to examine variations of those associations across subgroups by age, sex, smoking, alcohol drinking, and SAPS II. The significance of differences between the categories within subgroups was examined by Z-test. Sensitivity analysis was conducted to assess the robustness of the results by (1) varying the period of air pollutant exposure from 3 to 12 months, and (2) replacing the SAPS II by the number of injured organs as a covariate adjusted in the model. The number of injured organs (≤ 2, > 2; halved by the median number of patient’s injured/dysfunctional organs) was used as another measure of severity in critically ill patients [19]. According to the organ dysfunctions in the sequential organ failure assessment (SOFA), six organs were selected with their dysfunctions defined as follows: PaO_2_/FiO_2_ < 400 mmHg (respiratory system); Glasgow Coma Scale score ≤ 14 (nervous system); mean arterial pressure < 70 mm/Hg or administration of vasopressors required (cardiovascular system); bilirubin ≥ 1.2 mg/dl (liver); platelets < 150 × 10^3^/ml (blood system); and creatinine ≥ 1.2 mg/dl (kidney) [20, 21]. Weighted quantity sum (WQS) regression analysis was used to estimate the joint effect of the six air pollutants on mortality risk and relative contribution (i.e., weight) of each air pollutant to the joint effect [22]. All statistical analyses were performed in R (version 4.3.0). The level of significance was set at 0.05, and all tests were 2-sided.

## Results

### Characteristics of the study subjects

**Fig. 1:**
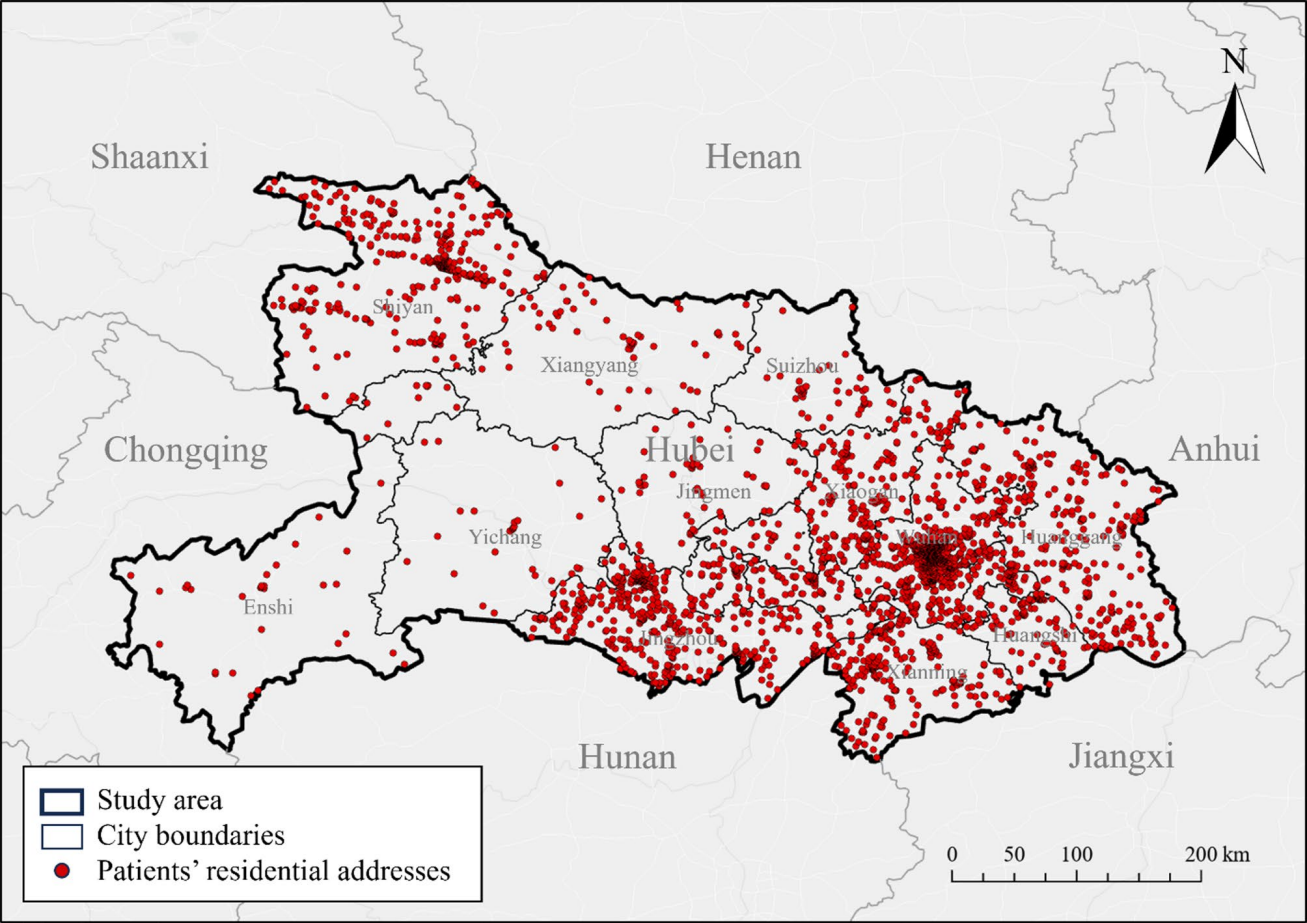
Spatial distribution of the patients involved in this study

The patients were excluded if (1) having incomplete residential addresses (*n* = 65); (2) being perioperative patients (*n* = 29); (3) being hospitalized by accidents (e.g., falling from a height, car accidents, suicide, self-injury) (*n* = 246). After exclusion, 7,222 living across the entire province were included in the final analysis (Fig. 1).

About 43.2% (3,123) of them were aged < 65, with 64.4% (4,654) being male (Table 1). About 27.1% (1,955) had smoking habits and 18.9% (1,366) had alcohol drinking habits. More than half of them were hospitalized ≤ 6 days (53.1%) and more than half of them had a SAPS II of > 33 (51.5%). The mortality rate within 28 days after ICU admission among the critically-ill patients was 39.1% (2,821).

**Table 1:** Characteristics of the patients included in this study

Variables	Number (%), Mean ± SD, or Median [IQR]			*p*-value^a^
	All (*n* = 7,222)	Survival (*n* = 4,401)	Death (*n* = 2,821)	
***Sex***				< 0.001
Male	4,654 (64.4)	2,738 (62.2)	1,916 (67.9)	
Female	2,568 (35.6)	1,663 (37.8)	905 (32.1)	
***Age*** (year)				< 0.001
< 65	3,123 (43.2)	2,079 (47.2)	1,044 (37.0)	
≥ 65	4,099 (56.8)	2,322 (52.8)	1,777 (63.0)	
***Smoking***				0.008
Yes	1,955 (27.1)	1,240 (28.2)	715 (25.3)	
No	5,267 (72.9)	3,161 (71.8)	2,106 (74.7)	
***Alcohol drinking***				0.052
Yes	1,366 (18.9)	864 (19.6)	502 (17.8)	
No	5,856 (81.1)	3,537 (80.4)	2,319 (82.2)	
***Number of injured organs***				< 0.001
≤2	3,185 (44.1)	2,326 (52.9)	859 (30.5)	
>2	4,037 (55.9)	2,075 (47.1)	1,962 (69.5)	
***Duration of hospitalization*** (day)				< 0.001
≤6	3,833 (53.1)	2,157 (49.0)	1,676 (59.4)	
>6	3,389 (46.9)	2,244 (51.0)	1,145 (40.6)	
***SAPS II***				< 0.001
≤33	3,504 (48.5)	2,780 (63.2)	723 (25.6)	
>33	3,718 (51.5)	1,621 (36.8)	2,098 (74.4)	
***Air pollutants***				
PM_2.5_ (µg/m^3^)	38.5 ± 13.4	37.7 ± 12.6	39.8 ± 14.5	< 0.001
	35.7 [18.7]	35.9 [19.1]	35.6 [17.6]	0.477
PM_10_ (µg/m^3^)	62.2 ± 21.4	61.2 ± 19.9	63.7 ± 23.5	< 0.001
	60.5 [27,5]	65.3 [12.0]	66.1 [15.6]	0.050
NO_2_ (µg/m^3^)	27.4 ± 11.5	26.1 ± 11.0	29.5 ± 12.0	< 0.001
	26.8 [19.6]	25.7 [18.7]	29.7 [20.6]	< 0.001
SO_2_ (µg/m^3^)	9.2 ± 2.2	9.2 ± 2.1	9.3 ± 2.3	0.180
	8.7 [2.7]	8.7 [2.7]	8.8 [2.6]	0.166
O_3_ (µg/m^3^)	60.7 ± 13.0	60.1 ± 12.5	61.5 ± 13.8	< 0.001
	61.2 [19.7]	60.6 [19.2]	61.9 [20.2]	< 0.001
CO (mg/m^3^)	0.8 ± 0.1	0.8 ± 0.1	0.9 ± 0.1	< 0.001
	0.9 [0.2]	0.8 [0.2]	0.9 [0.1]	< 0.001

^a^p-values tested the significance of differences in each variable between the participants who survived and died, and were calculated from χ [2] tests for categorical variables or *t*-tests for continuous variables (Mann-Whitney *U* tests for variables denoted by Median [IQR])

CO, carbon monoxide; IQR, interquartile-range; NO_2_, nitrogen dioxide; O_3_, ozone; PM_2.5_, particulate matter with an aerodynamic diameter of < 2.5 μm; PM_10_, particulate matter with an aerodynamic diameter < 10 μm; SO_2_, sulfur dioxide

The median concentrations of air pollutants were 60.5 [IQR: 48.6–76.1] µg/m^3^ for PM_10_, 61.2 [51.1–70.8] µg/m^3^ for O_3_, 26.8 [18.3–37.9] µg/m^3^ for NO_2_, 35.7 [29.5–48.2] µg/m^3^ for PM_2.5_, 8.7 [7.6–10.3] µg/m^3^ for SO_2_, and 0.9 [0.7–0.9] mg/m^3^ for CO (Table 1). The exposure levels of all air pollutants among the deaths were significantly higher than among those alive, except for SO_2_ (9.2 ± 2.1 vs. 9.3 ± 2.3 µg/m^3^, *p* = 0.180).

### Associations between air pollutants and mortality risk

In crude models, a higher risk of mortality was associated with the each unit increase in the concentration of PM_2.5_ (OR = 1.005 [95% CI: 1.001, 1.008]), PM_10_ (OR = 1.001 [1.000, 1.003]), NO_2_ (OR = 1.021 [1.016, 1.025]), SO_2_ (OR = 1.027 [1.006, 1.050]), O_3_ (OR = 1.008 [1.004, 1.012]), and CO (OR = 3.895 [2.738, 5.542]) (Table 2). After adjusting for demographic and lifestyle covariates, especially SAPS II, the associations remained significant PM_2.5_ (OR = 1.007 [1.003, 1.011]), PM_10_ (OR = 1.002 [1.000, 1.004]), NO_2_ (OR = 1.020 [1.015, 1.024]), SO_2_ (OR = 1.025 [1.001, 1.050]), O_3_ (OR = 1.005 [1.001, 1.009]), and CO (OR = 4.336 [2.952, 6.457]).

**Table 2:** Associations between each unit (interquartile range) increase in the concentration of air pollutants and the mortality risk

Variables	OR (95% CI)			
	Crude	Model 1	Model 2	Model 3
Each unit risk increase				
PM_2.5_ (µg/m^3^)	1.005^**^ (1.001, 1.008)	1.004^*^ (1.000, 1.007)	1.003^*^ (1.000, 1.007)	1.007^***^ (1.003, 1.011)
PM_10_ (µg/m^3^)	1.001^*^ (1.000, 1.003)	1.001^*^ (1.000, 1.003)	1.001^*^ (1.000, 1.002)	1.002^*^ (1.000, 1.004)
NO_2_ (µg/m^3^)	1.021^***^ (1.016, 1.025)	1.017^***^ (1.013, 1.022)	1.017^***^ (1.013, 1.021)	1.020^***^ (1.015, 1.024)
SO_2_ (µg/m^3^)	1.027^*^ (1.006, 1.050)	1.020 (0.998, 1.043)	1.019 (0.997, 1.041)	1.025^***^ (1.001, 1.050)
O_3_ (µg/m^3^)	1.008^***^ (1.004, 1.012)	1.009^***^ (1.005, 1.012)	1.009^***^ (1.005, 1.012)	1.005^***^ (1.001, 1.009)
CO (mg/m^3^)	3.895^***^ (2.738, 5.542)	3.149^***^ (2.202, 4.502)	3.121^**^ (2.180, 4.468)	4.336^***^ (2.952, 6.457)
Each interquartile-range risk increase				
PM_2.5_ (µg/m^3^)	1.090^**^ (1.023, 1.163)	1.068^*^ (1.001, 1.140)	1.065^*^ (1.001, 1.137)	1.141^***^ (1.061, 1.226)
PM_10_ (µg/m^3^)	1.022^*^ (1.001, 1.045)	1.008^*^ (1.001, 1.021)	1.005^*^ (1.001, 1.018)	1.032^*^ (1.001, 1.067)
NO_2_ (µg/m^3^)	1.490^***^ (1.375, 1.614)	1.402^***^ (1.291, 1.521)	1.394^***^ (1.284, 1.513)	1.466^***^ (1.340, 1.604)
SO_2_ (µg/m^3^)	1.076^*^ (1.015, 1.140)	1.056 (0.996, 1.120)	1.051 (0.991, 1.115)	1.069^*^ (1.002, 1.140)
O_3_ (µg/m^3^)	1.173^***^ (1.092, 1.260)	1.186^***^ (1.104, 1.275)	1.184^***^ (1.102, 1.273)	1.111^**^ (1.027, 1.202)
CO (mg/m^3^)	1.261^***^ (1.188, 1.339)	1.216^***^ (1.144, 1.293)	1.214^***^ (1.142, 1.291)	1.286^***^ (1.203, 1.375)

Model 1: Adjusted for sex and age; Model 2: Additionally adjusted for smoking and alcohol drinking upon Model 1; Model 3: Additionally adjusted for SAPS II upon Model 2

^*^p < 0.05; ^**^p < 0.005; ^***^p < 0.001

CO, carbon monoxide; NO_2_, nitrogen dioxide; O_3_, ozone; PM_2.5_, particulate matter with an aerodynamic diameter of < 2.5 μm; PM_10_, particulate matter with an aerodynamic diameter < 10 μm; SO_2_, sulfur dioxide

### Variations of the associations between air pollutants and mortality risk

**Fig. 2:**
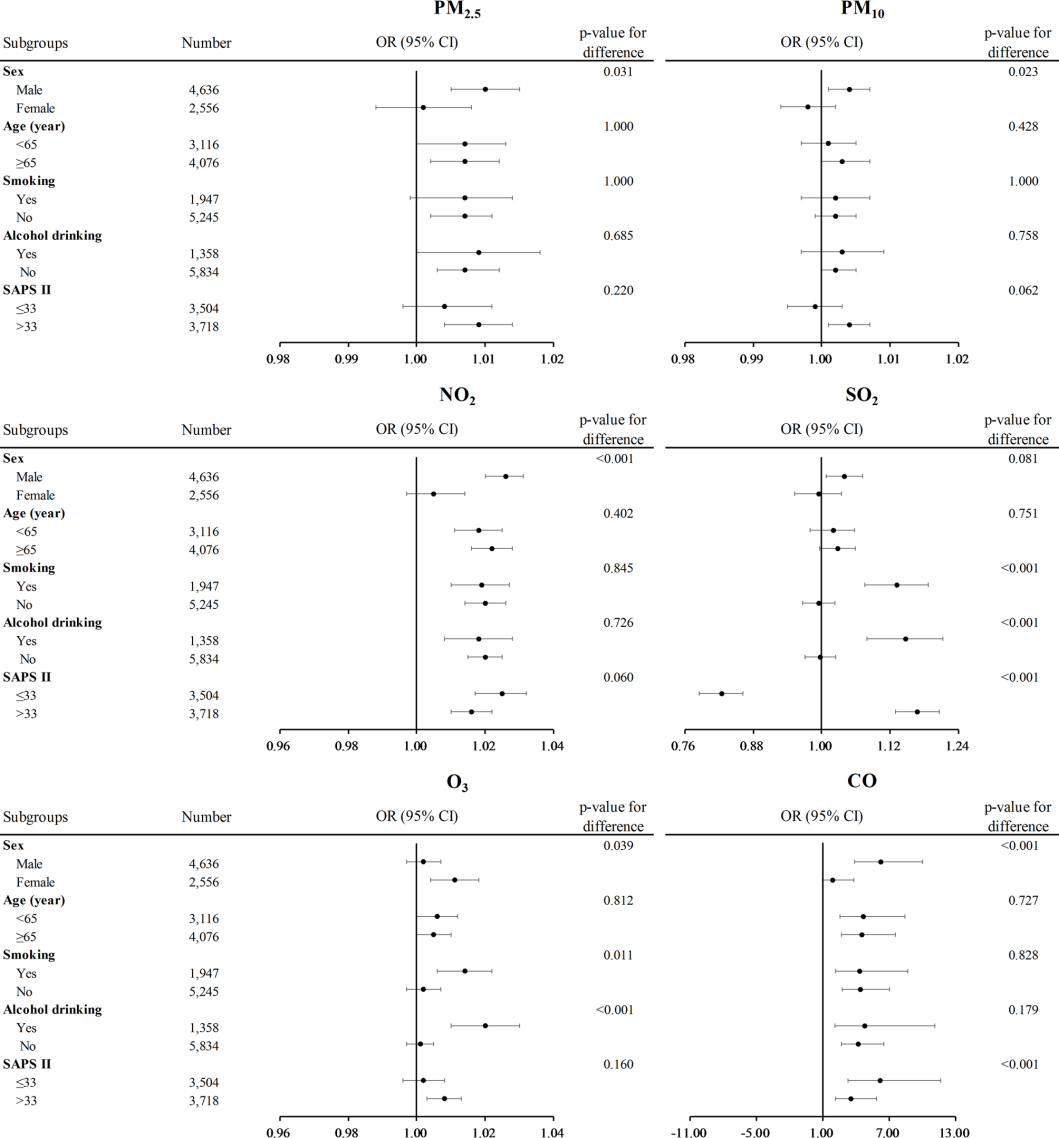
Forest plot of the associations between air pollutants and mortality risk in the subgroups. All covariates (age, sex, smoking, alcohol drinking, and SAPS II) except for the one used for stratification were adjusted. A Z-test was used to test for significance of differences in odds ratio (OR) estimates across categories within subgroups

The mortality risks corresponding to each unit increase in the concentrations of PM_2.5_, PM_10_, NO_2_, SO_2_, O_3_, and CO varied across subgroups (Fig. 2). For example, the higher mortality risks were observed in males for PM_2.5_ (OR = 1.010 [1.005, 1.015]), PM_10_ (OR = 1.004 [1.001, 1.007]), NO_2_ (OR = 1.026 [1.021, 1.032]), and CO (OR = 6.224 [3.867, 10.019]), whereas a stronger association of O_3_ with mortality risk was found in females (OR = 1.011 [1.004, 1.018]). The stronger associations of SO_2_ and O_3_ with mortality risk were also observed in smokers (SO_2_: OR = 1.132 [1.078, 1.189], O_3_: OR = 1.014 [1.006, 1.022]) and alcohol drinkers (SO_2_: OR = 1.147 [1.082, 1.215], O_3_: OR = 1.020 [1.010, 1.029]). The associations between most of the air pollutants and mortality risk were stronger in those with a SAPS II of > 33 (SO_2_: OR = 1.168 [1.130, 1.207], and CO: OR = 3.557 [2.165, 5.843]).

The main results remained robust after varying the period of exposure to air pollutants from 3 to 12 months (Table 3). Note that, the association between a unit increase of SO_2_ and mortality risk was not significant when the period of exposure was from 9 months to 12 months or after replacing SAPS II by the number of injured organs as a covariate adjusted in the model (Table 3).

**Table 3:** Associations between each unit increase in the concentration of air pollutants and the mortality risk over different periods of exposure and by adjusting for the number of injured organs rather than SAPS II

Variables	OR (95% CI)			
	3-month exposure	9-month exposure	12-month exposure	Adjusting for the number of injured organs
PM_2.5_ (µg/m^3^)	1.009^***^ (1.006, 1.012)	1.005^*^ (1.001, 1.010)	1.017^***^ (1.012, 1.022)	1.004^*^ (1.001, 1.008)
PM_10_ (µg/m^3^)	1.004^***^ (1.002, 1.006)	1.002^*^ (1.000, 1.005)	1.005^**^ (1.002, 1.008)	1.001^*^ (1.000, 1.003)
NO_2_ (µg/m^3^)	1.021^***^ (1.017, 1.025)	1.019^***^ (1.014, 1.023)	1.023^***^ (1.019, 1.028)	1.023^***^ (1.019, 1.027)
SO_2_ (µg/m^3^)	1.024^*^ (1.002, 1.047)	0.994 (0.969, 1.019)	0.983 (0.958, 1.009)	1.008 (0.985, 1.030)
O_3_ (µg/m^3^)	1.002 (0.999, 1.005)	1.012^***^ (1.007, 1.017)	1.010^***^ (1.005, 1.015)	1.008^***^ (1.004, 1.012)
CO (mg/m^3^)	4.333^***^ (3.026, 6.205)	3.885^***^ (2.558, 5.901)	8.656^***^ (5.598, 13.384)	4.253^***^ (2.941, 6.151)

All models adjusted for sex, age, smoking, alcohol drinking, and SAPS II or the number of injured organs

^*^p < 0.05; ^**^p < 0.005; ^***^p < 0.001

CO, carbon monoxide; NO_2_, nitrogen dioxide; O_3_, ozone; PM_2.5_, particulate matter with an aerodynamic diameter of < 2.5 μm; PM_10_, particulate matter with an aerodynamic diameter < 10 μm; SAPS II, simplified acute physiology score; SO_2_, sulfur dioxide

### Relative contributions of air pollutants to their joint health effect

**Fig. 3:**
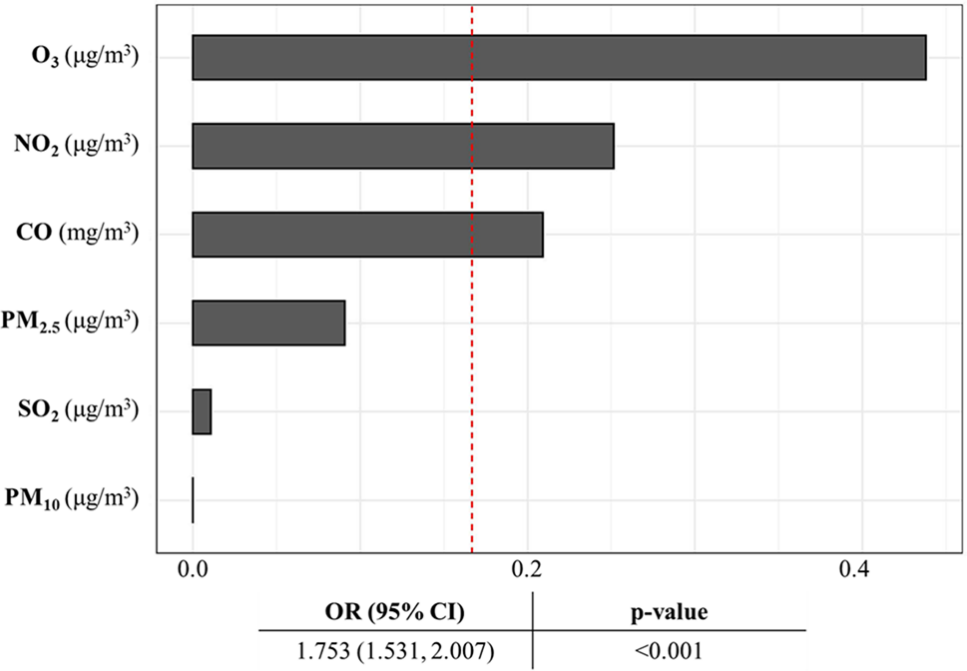
Relative contribution of each air pollutant to their joint effect on the mortality risk, after adjusting for all covariates. Age, sex, smoking, alcohol drinking, and SAPS II were adjusted in the model

The contributions of the six air pollutants to their joint effect on mortality risk, estimated as the effect of each one-quartile increase in the mixture of the six air pollutants (OR = 1.753 [1.531, 2.007]), were calculated (Fig. 3). The largest contribution was from O_3_ (43.8%), followed by NO_2_ (25.1%), CO (20.9%), PM_2.5_ (9.1%), SO_2_ (1.0%), and PM_10_ (0.1%).

## Discussion

This is the first study examining the associations of multiple air pollutants with and their relative contributions to mortality risk among critically ill patients. It revealed that PM_2.5_, PM_10_, NO_2_, O_3_, and CO were positively associated with mortality risk. O_3_ was the main contributor to the joint effect of air pollutants on mortality risk. Stronger associations were mainly observed in males, smokers, alcohol drinkers, and those with a SAPS II of > 33. The associations were robust when varying the period of exposure to air pollutants from 3 to 12 months.

The associations between air pollutants and mortality risk found in this study were similar to some previous results in non-ICU contexts. For example, a nationwide cohort study in China found that an increase in O_3_ concentration was associated with the increased cardiovascular mortality risk among 96,955 adults [23]. Another Chinese study found that an increase in SO_2_ concentration was associated with the increased mortality risk among 11,199 older adults [24]. A Korean study showed that higher concentrations of PM_2.5_ and CO were associated with the higher mortality risk among 32,949 chronic kidney disease patients from the three hospitals [25]. However, the severity of comorbidities in patients that could affect their mortality risk has not been controlled in previous studies, which may make the associations between air pollutant exposure and mortality risk overestimated. In this study, we controlled for not only sociodemographic and clinical characteristics but also the severity of disease, measured as the SAPS II and the number of injured organs, aiming to rule out potential effects of disease severity on mortality risk. The association between PM_2.5_ and mortality risk found in this study was also consistent with the one found in Australia, where an increase in PM_2.5_ concentration over the three days prior to hospital admission was associated with the increased mortality in ICU patients in Australia [26]. Such consistencies suggest that both short-term and long-term exposure to PM_2.5_ may matter regarding the impact on mortality risk of ICU patients, which highlighted the importance of air quality regulations and targeted interventions to this vulnerable population.

O_3_ was found to be the largest contributor to the negative health effect of air pollutants in critically ill patients. In some previous epidemiological studies, exposure to a higher level of O_3_ was associated with the increased mortality risk [27–29]. However, few studies investigated the role of O_3_ in the joint effect of air pollutants, among which the contribution of O_3_ to the joint effect varied [30, 31]. For example, a 22-year study of general populations in northern China found that PM_2.5_ instead of O_3_ was the largest contributor in the joint effect of air pollutant mixture on mortality risk [30]. A multi-city study in the US also found that PM_2.5_ had a larger contribution to all-cause mortality than O_3_ and NO_2_ [31]. However, it was suggested that the importance of O_3_ in the health effect of air pollutants should not be neglected [30]. On one hand, O_3_ is one of the most toxic air pollutants, causing oxidative stress, inflammation of the airways and premature deaths in the general population [32]. On the other hand, other air pollutants, such as PM_2.5_, CO, and NO_2_, can react with free radicals (e.g., HO_2_, volatile organic compounds) in the troposphere, promoting more O_3_ production [33–35]. Furthermore, some daily products (e.g., pesticides, coatings, printing inks, and personal care products), vehicular exhaust and even natural vegetation can also emit some volatile organic compounds (e.g., isoprene), which could contribute to O_3_ formation and may increase mortality risk [36]. 

A stronger association between air pollutants and mortality risk was observed in patients with higher SAPS II. One possible explanation is that patients with higher SAPS II may have more injured organs [37]. Some studies found that air pollutants might mainly worsen lung injuries and have limited influences on other organs [38, 39]. However, some findings also suggested that air pollutants could traverse the alveoli and affect other organs throughout the body, resulting in deterioration of organs such as liver and kidneys, which may increase the risk of mortality [40–42]. 

There are three main strengths in this study. First, for the first time, this study examined associations between exposure to the common air pollutants and mortality risk of critically ill patients. Moreover, using WQS regression to avoid the problem of collinearity between air pollutants, this study further estimated the joint effect of multiple air pollutants on mortality risk and their respective contributions to the joint effect [43]. This is crucial for understanding mechanisms underlying health effects of air pollutants and providing targeted strategies to counteract those adverse health effects. Second, the severity of comorbidities in patients affecting their mortality risk were known in this study, hence increasing the reliability of the results. It has not been considered in relevant epidemiological studies before, which may make the associations between air pollutant exposure and mortality risk of the similar populations overestimated in previous studies. Third, this is the first epidemiological study using the known highest-resolution air pollutant products to estimate the exposure to air pollutants, generated by the newly developed multi-output LightGBM model. The model integrates the multi-output regression algorithm with the LightGBM framework and, regarding runtime efficiency, outperforms the well-known traditional machine learning models, such as Extreme Gradient Boosting and Extremely Randomized Trees [16]. 

Some limitations in this study need to be acknowledged. First, the covariates adjusted in the models may not be sufficient. We have only adjusted for some sociodemographic and behavioral features with strong theoretical foundations to affect the mortality risk, and some clinical features closely related to the mortality risk. Some important potential covariates were not adjusted in the models due to lack of data, such as socioeconomic status, which would be collected or indirectly estimated in future efforts. Also, COVID-19 may affect the results of this study as it could affect both the levels of air pollutants and health conditions of ICU patients. However, the study period chosen in this study has minimized the influences of COVID-19. Perhaps a few ICU patients involved in this study were still affected by COVID-19 or other diseases/comorbidities (perhaps linked to chronic air pollutant exposure), which, however, should have been reflected as injured organs that were adjusted in the model. Second, the pre-admission exposure to air pollutants was measured as the modeled concentrations of air pollutants over patients’ residential addresses, which may not accurately capture the spatial and temporal variability of air pollutant concentrations. Also, as they may move around during the period before going to ICU, only considering residential locations could lead to exposure misclassification [44, 45]. However, compared to other air pollution epidemiological studies of the general populations who usually have a larger mobility capacity [46, 47], our results are quite reliable owing to the limited mobility of critically ill patients. In addition, the measure of O_3_ exposure in this study was different from the way of reporting the level of O_3_ in the World Health Organization Global Air Quality Guidelines, which, for the purpose of monitoring the long-term changing trend of O_3_ concentration, was reported the average of daily maximum 8-hour mean O_3_ concentration in the six consecutive months with the highest 6-month running-average O_3_ concentration. The average daily concentration of O_3_ over a given study period has been a widely used measure of O_3_ exposure in existing epidemiological studies; [48, 49] moreover, in this study, the patients’ personal window of exposure in the main analysis was 6 months, and some of them may not include the warm season. Future research on longer-term (e.g., > 1 year) exposure to O_3_ concentration may consider using different measures of O_3_ concentration. Third, considering that imputing missing residential addresses for estimating the patients’ exposure to air pollutants may cause uncertainties, those with incomplete addresses were excluded, which may cause biases to some extent. However, due to a small number of patients with incomplete residential addresses in this study (< 20), the biases and their influences should be limited. In future research with more patients with incomplete data, other missing or incomplete characteristics than residential addresses (e.g., basic sociodemographic features) would be imputed to reduce the biases. Fourth, the study subjects were mainly from authoritative hospitals in central China. Critically ill patients are often admitted to their ICUs of such hospitals, thus leading to the higher mortality in them than in general hospitals reported in some public health studies (e.g., 39% in this study). A previous study has found that the average mortality of ICU patients could be 11-42% [50, 51]. Also, the sample size and study area included in this study are limited. Although this may affect the generalizability of the results to larger populations and regions, the relatively high levels of PM_2.5_ concentration in the study area could have increased the ability to highlight the effects of air pollutants on the outcome. Given that the Hubei Province Medical Treatment Alliance is expanding and recruiting more participating hospitals and patients, more research with a larger sample size in more hospitals and regions is expected to further consolidate and strengthen the generalizability of these findings.

## Conclusions

Long-term exposure to higher concentrations of air pollutants was associated with a higher mortality risk among critically ill patients. The association varied by age, sex, alcohol drinking status, diseased organ, and SAPS II. Among the common air pollutants, O_3_ contributed most to the mortality risk, followed by NO_2_, CO, PM_2.5_, SO_2_, and PM_10_. The findings would help multiple stakeholders better understand health effects of air pollutants on critically ill patients and also serve as justifications for facilitate environmental justice and health equity. Future research with different measurements of confounding factors, more accurate assessments of exposure, and a larger sample size and study area, would help consolidate the findings of this study and advance this research area.

## Data Availability

The datasets from this study are held in coded form, and legal data sharing agreements prohibit the authors from making the dataset publicly available. Access to individual deidentified participant data (including data dictionaries) may be granted to those who send a reasonable request with specific data needs, analysis plans, and dissemination plans to the corresponding authors.
